# Diversity and prevalence of *Leucocytozoon* in black flies (Diptera: Simuliidae) of Thailand

**DOI:** 10.1186/s13071-024-06567-0

**Published:** 2024-11-19

**Authors:** Waraporn Jumpato, Wannachai Wannasingha, Chavanut Jaroenchaiwattanachote, Ronnalit Mintara, Komgrit Wongpakam, Peter H. Adler, Pairot Pramual

**Affiliations:** 1 Department of Biology, Faculty of Science, Mahasarakham University, Kantharawichai District, Mahasarakham, 44150 Thailand; 2 Center of Excellence in Biodiversity Research, Mahasarakham University, Mahasarakham, 44150 Thailand; 3 Walai Rukhavej Botanical Research Institute, Mahasarakham University, Kantharawichai District, Mahasarakham, 44150 Thailand; 4 Department of Plant and Environmental Sciences, Clemson University, Clemson, SC 29634-0310 USA

**Keywords:** Haemosporidian parasites, *Simulium*, Leucocytozoonosis

## Abstract

**Background:**

Leucocytozoonosis, a parasitic disease of birds, is caused by haemosporidian protozoan parasites of the genus* Leucocytozoon*, which infect diverse avian species, including poultry. These parasites are transmitted by several black fly species, but knowledge of the factors determining the diversity and prevalence in these vectors, which is crucial for fully understanding disease epidemiology, is largely unexplored. In this study, we investigated factors associated with the prevalence and diversity of *Leucocytozoon* species in black flies from Thailand.

**Methods:**

Adults of two black fly taxa (*Simulium asakoae* Takaoka and Davies complex and *S. khelangense* Takaoka, Srisuka and Saeung) were collected using sweep nets at nine locations in northern and northeastern regions of Thailand. Specimens were identified morphologically and the results corroborated by DNA barcoding. Molecular methods using specific primers for amplification of the mitochondrial cytochrome* b* (cyt *b*) gene of *Leucocytozoon* were used to detect the parasite in black flies. Species and lineages of *Leucocytozoon* were determined using the MalAvi database of malaria parasites and related haemosporidians in avian hosts. Regression analysis was used to examine relationships between *Leucocytozoon* diversity and prevalence, black fly abundance and habitat characteristics.

**Results:**

A total of 11,718 adult black flies were collected, of which 4367 were members of the *S. asakoae* complex and 7351 were *S. khelangense*. For molecular detection of *Leucocytozoon*, we randomly selected 300 individual female black flies of the *S*. *asakoae* complex and 850 females of *S*. *khelangense* pooled into groups of five individuals (= 170 pools). A total of 34 of the 300 specimens of the *S. asakoae* complex and 118 of the 170 pools of *S*. *khelangense* were positive for *Leucocytozoon*. Fifty-four lineages (haplotypes) were identified, all of which belonged to those reported in domestic chickens, *Gallus gallus*, with one exception that was identified in *S. khelangense* and found to be closely related to the *Leucocytozoon* lineages reported in owls; this is the first record of the latter lineage in Asian black flies. Among these haplotypes, nine and 45 were exclusively found in the *S. asakoae* complex and *S. khelangense*, respectively. No lineage was shared between these black fly taxa. Analysis of similarity (ANOSIM) revealed significant *Leucocytozoon* lineage composition between the two black flies. Phylogenetic analysis found that *Leucocytozoon* lineages in the *S. asakoae* complex and *S. khelangense* are largely isolated, agreeing with the ANOSIM result. The overall prevalence of *Leucocytozoon* in the *S. asakoae* complex was 11.3% and ranged from 9% to 13% in each collection. *Leucocytozoon* prevalence in *S. khelangense* was 21%, varying from 13% to 37% in each collection. The Shannon H′ index indicated greater *Leucocytozoon* diversity in *S. khelangense* (H′ = 3.044) than in the *S. asakoae* complex (H′ = 1.920). Regression analysis revealed that *Leucocytozoon* diversity was positively related to black fly abundance and negatively related to maximum air temperature.

**Conclusions:**

The results of this study show that the prevalence and diversity of *Leucocytozoon* lineages in the *S*. *asakoae* complex and *S. khelangense* from Thailand were associated with the abundance of these black flies and with air temperature. The *Leucocytozoon* lineages identified also showed some degree of black fly taxon specificity, possibly related to different abundance peaks of these vectors. The environmental conditions that favor the development of black flies are possibly a driver of *Leucocytozoon* prevalence, diversity and vector–parasite co-evolution.

**Graphical Abstract:**

**Figure:**
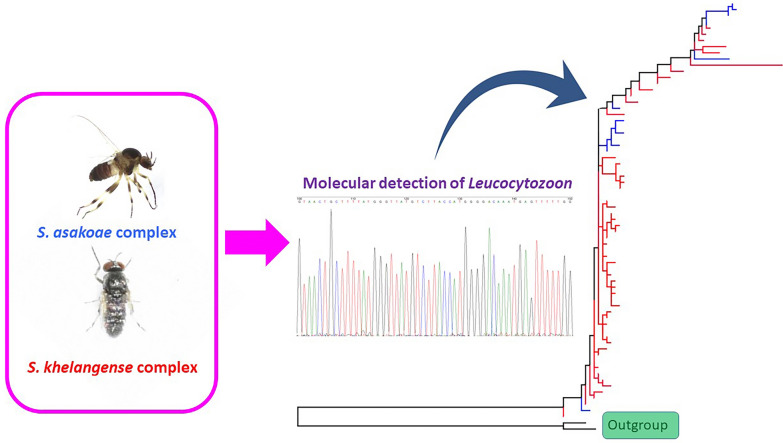

**Supplementary Information:**

The online version contains supplementary material available at 10.1186/s13071-024-06567-0.

## Background

Haemosporidian parasites of the genus *Leucocytozoon* infect diverse avian hosts globally [1]. At least 45 morphological species are currently recognized, but much greater diversity has been documented (1542 lineages in the MalAvi database; http://130.235.244.92/Malavi/, accessed 31 October 2024) based on the mitochondrial cytochrome* b* (cyt* b*) sequence [2]. *Leucocytozoon* infections can cause a disease known as leucocytozoonosis, which is typically more severe in poultry than in wild birds [1, 3, 4]. Leucocytozoonosis can reduce growth rate and egg production and even lead to death, causing significant economic losses to the poultry industry [3].

The majority of *Leucocytozoon* species are transmitted by black flies (Diptera: Simuliidae), with the exception of one species, *L*. (*Akiba*) *caulleryi*, which is transmitted by biting midges (Ceratopogonidae: *Culicoides*) [5, 6]. To date, 48 black fly species are known to be vectors or potential vectors of *Leucocytozoon* [3, 6–13], but knowledge of these vector species represents only a fraction of the vast diversity of *Leucocytozoon* genetic lineages [14]. Similarly, among the 2407 extant species of the world’s black flies [15], numerous species have not yet been investigated for their involvement in parasite transmission, and most studies so far have focused on black flies in North America [3].

Vectors are crucial components of vector-borne disease epidemiology. Therefore, knowledge of the factors related to the transmission of pathogens by vectors is central to effective disease control and prevention. Key factors in the successful transmission of haemosporidian parasites to susceptible hosts are the environmental conditions that determine vector abundance and distribution, both of which can influence the prevalence and diversity of the parasites [16]. Factors that relate to these vector population characteristics include rainfall, elevation, season and temperature [16]. For example, a high prevalence of *Leucocytozoon* in wild birds in the highlands of Neotropical Colombia is associated with elevation [9]. Seasonal changes in temperature affect the emergence of black fly vectors and are related to the prevalence of *Leucocytozoon* in vertebrate hosts [17]. However, factors associated with the prevalence and diversity of *Leucocytozoon* in black fly vectors remain largely unexplored [16].

In Thailand, the prevalence of *Leucocytozoon* is high in domestic chickens (up to 89%) [18] compared with wild bird species (2–8%) [19, 20]. At least three black fly taxa (*Simulium asakoae* Takaoka and Davies complex, *S. chumpornense* Takaoka and Kuvangkadilok and *S. khelangense* Takaoka, Srisuka and Saeung) are potential vectors of [10, 13, 21, 22], and there is some degree of association between vector species and parasitic lineages [13]. The prevalence rates of *Leucocytozoon* in black flies vary geographically and seasonally [10, 13, 22]. For example, in one study, the majority (91%) of *Leucocytozoon* detected in *S*. *khelangense* (= *S. chumpornense* in Pramual et al. [22]) were from black flies collected during the dry season (March–May) and at the beginning of the rainy season (June), with only 9% collected in the late rainy season (September) [22]. This finding reinforces the possibility that environmental factors related to season might affect the prevalence and distribution of *Leucocytozoon*.

In the study reported here we investigated the prevalence and diversity of *Leucocytozoon* in two black fly taxa, the *S*. *asakoae* complex and *S*. *khelangense*. Adult black flies were collected from various localities that had not been investigated previously in Thailand. We tested whether the diversity and prevalence of *Leucocytozoon*, based on mitochondrial cyt* b* lineages, are associated with black fly abundance and habitat characteristics. We also explored whether *Leucocytozoon* lineages are associated with specific black fly taxa.

## Methods

### Specimen collection and identification

**Fig. 1:**
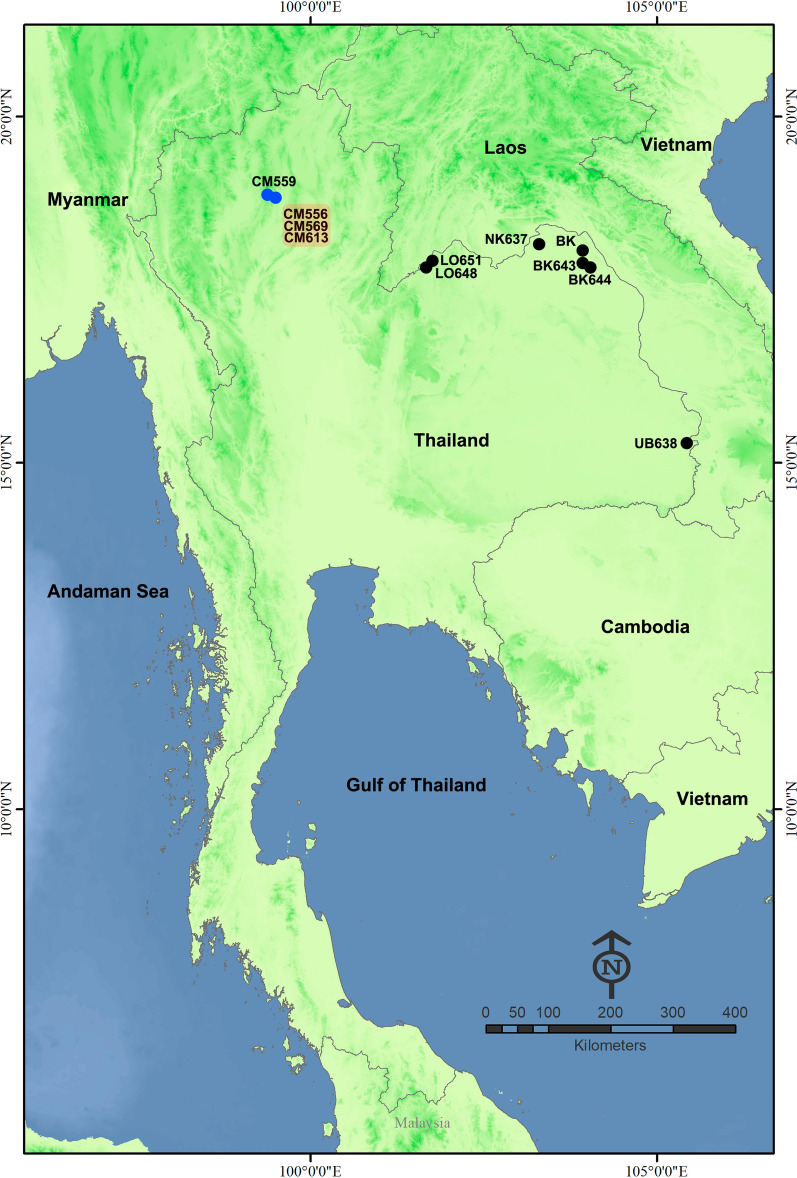
Sampling locations of black flies of the *Simulium asakoae* complex (blue-filled circles) and *Simulium khelangense* (black-filled circles, used for molecular detection of *Leucocytozoon* parasites. Details on each sampling site are given in Table 1

Eleven collections of wild adult black flies were carried out at nine sampling sites in northern and northeastern Thailand from August 2019 to February 2024 (Table 1; Fig. 1). Specimens were collected with a sweep net (39-cm-diameter hoop with a 3-part telescopic handle and total extended length of 120 cm). The sweep net was moved back and forth in a figure eight motion 0.5–2.0 m above the ground. Specimens were collected during times when adult black flies were actively searching for hosts, specifically early in the morning (06:00–08:00 h) or late afternoon (16:00–18:00 h) [23, 24]. Duration of collection times for each sampling location varied from 30 min to 4 h and involved between one and four collectors each collection. The adult black flies collected were placed in plastic vials containing 80% (v/v) ethanol and stored at − 20 °C until they were sorted out from other collected insects under a stereomicroscope in the laboratory. Only female black flies were used in subsequent analyses. The specimens were examined under a stereomicroscope for the presence of a blood meal and then used for host blood-source identification. Species were identified morphologically using known keys and descriptions of black flies in Thailand [25, 26].

**Table 1:** Sampling locations, black fly taxa, number of specimens used for molecular detection of *Leucocytozoon* parasites and number of positive detections

Species	Location (code)	Coordinates (N E)	Elevation (m a.s.l.)	Collection date (season)^a^	Number of specimens tested for *Leucocytozoon* (no. positive)	Prevalence (%)
*Simulium asakoae* complex	Road to Ban Pang Bong, Doi Saket, Chiang Mai (CM559)	18.988295 99.339096	1013	24 Aug 2019 (R)	34 (3)	9
	Ban Pang Bong, Doi Saket, Chiang Mai (CM556)	18.985475 99.335629	1080	23 Aug 2019 (R)	66 (6)	9
	Ban Pang Bong, Doi Saket, Chiang Mai (CM596)	18.985475 99.335629	1080	25 Jan 2020 (C)	100 (12)	12
	Ban Pang Bong, Doi Saket, Chiang Mai (CM613)	18.985475 99.335629	1080	10 Feb 2023 (C)	100 (13)	13
Subtotal for *S. asakoae* complex					300 (34)	11.3
*Simulium khelangense*	Ban Non Du, Rattanawapi, Nong Khai (NK637)	18.193845 103.321423	150	31 Dec 2023 (C)	50 pools (31)	18
	Khong Chiam, Ubon Ratchathani (UB638)	15.316529 105.512555	120	13 Jan 2024 (C)	10 pools (10)	NA
	Bueng Khong Long, Bueng Kan (BK)	18.047119 103.980453	168	21 Jan 2024 (C)	40 pools (20)	13
	Phu Wua, Bueng Kan (BK644)	18.234556 103.962278	180	20 Jan 2024 (C)	20 pools (18)	37
	Ban Bung Khla, Bueng Kan (BK643)	18.263339 103.982664	160	20 Jan 2024 (C)	10 pools (10)	NA
	Chiang Khan (1), Loei (LO648)	17.834947 101.616884	210	17 Feb 2024 (H)	20 pools (17)	32
	Chiang Khan (2), Loei (LO651)	17.907008 101.696557	220	18 Feb 2024 (H)	20 pools (12)	17
Subtotal for *S. khelangense*					170 pools (118)	21.1

^a^Season: R, Rainy; C, cold; H, hot

### Molecular analysis

#### PCR-based identification of black flies

Females of *S. khelangense* are difficult to identify based on morphological characteristics; therefore, we emplyed a molecular method based on cytochrome* c* oxidase I (COI) to facilitate species identification. Prior to processing for DNA extraction, specimens were dried at room temperature to evaporate the ethanol. DNA was extracted from individual specimens using the GF-1 Nucleic Acid DNA Extraction Kit (Vivantis Technologies Sdn. Bhd., Shah Alam, Selangor, Malaysia), following the manufacturer’s protocol. The primers LCO1490 and HCO2198 [27] were used to amplify an approximately 650-bp fragment of the COI gene. The PCR reaction conditions followed those of Tangkawanit et al. [28]. PCR products were stained with Novel Juice (GeneDireX, Taoyuan, Taiwan, Republic of China) and were checked by 1% agarose gel electrophoresis. Successful amplifications were then purified using the PureDirex PCR CleanUp & Gel Extraction Kit (Bio-Helix, Taiwan, Republic of China), following the manufacturer’s protocol. The purified PCR products were sequenced at ATCG Company Limited (Thailand Science Park, Pathumthani, Thailand) using the same primers as for PCR.

#### Host blood-meal identification

Among the 11,718 specimens collected, only four were blood engorged and all were *S. khelangense*. These specimens were used for molecular identification of the host blood source. DNA was extracted from the whole individual using the same method as for PCR-based identification of black flies. The vertebrate host blood mitochondrial cyt *b* was amplified using primers L14841 and H15149 [29], with PCR reaction conditions as described by Malmqvist et al. [30]. PCR product checking, purification and sequencing were as for the DNA barcoding study but the primers for cyt *b* were used for sequencing.

#### Molecular detection of *Leucocytozoon*

A total of 1150 female black flies were randomly selected for molecular detection of *Leucocytozoon*. Of these, 300 females of the *S*. *asakoae* complex were used individually and 850 females of *S*. *khelangense* were pooled into groups of five individuals each for a total of 170 pools. The DNA extraction method was the same as that used in the PCR-based identification of black flies. The nested PCR method described by Hellgren et al. [31] was used to amplify *Leucocytozoon* DNA in black flies. Specific primers (HaemNFI and HaemNR3 for the first round and HaemFL and HaemR2L for the second round PCR) were used to amplify an approximately 500-bp fragment of cyt* b* of the *Leucocytozoon* mitochondrial DNA [31]. The PCR reaction conditions followed those of Jumpato et al. [10]. Purification and sequencing of the PCR products used the same method as described for the COI gene but with the specific primers for *Leucocytozoon* for sequencing.

### Data analysis

Sequences of all genes were checked for quality using the “Edit/View sequencer file” option in MEGA X [32]. Three COI sequences (accession nos. PQ146532-PQ146534) were obtained from representative specimens identified morphologically as *S*. *khelangense*. These sequences were compared using the Basic Local Alignment Search Tool (BLAST); all had > 99% similarity with *S*. *khelangense*. The cyt* b* sequences (accession nos. PQ425370–PQ425372) of the host blood meals were successfully obtained from three of the four blood-engorged specimens. These sequences were compared with those of vertebrate cyt* b* in the National Center for Biotechnology Information (NCBI) GenBank, using BLAST. The vertebrate host was considered identified if sequence similarity was > 98%.

The cyt* b* sequences of *Leucocytozoon* parasites were checked and evaluated based on the sequence chromatogram. Only clear chromatograms, with no double peaks that might have indicated the possibility of co-infection, were used for further analysis. These sequences were submitted to NCBI GenBank (accession nos. PQ155266–PQ155417). Identification of *Leucocytozoon* lineages was performed using the BLAST method in the MalAvi database [14] (accessed 20 April 2024). A lineage was considered to be known if the sequence was a 100% match with any of those in the database; in this case, the lineage names reported in the MalAvi database were used. If the sequences obtained in the present study showed < 100% match (i.e., single-base differentiation), we considered it to be a new lineage. The new lineage sequences were then submitted to MalAvi and named following the database instructions by using the abbreviation of the host species name followed by a number [14]. Neighbor-joining (NJ) and maximum likelihood (ML) were used to examine genetic relationships between cyt *b* lineages of *Leucocytozoon* parasites found in the present study (54 lineages) plus those previously reported in black flies from Thailand (12 lineages) [10, 13, 21, 22]. Branch support was estimated using 1000 bootstrapping replications. The NJ and ML trees were inferred using MEGA X [32].

We used regression analysis to test the relationship between *Leucocytozoon* diversity, prevalence and environmental factors (minimum and maximum temperature). In addition to data obtained in the present study, we also included information in the regression analysis on the habitats and *Leucocytozoon* diversity and prevalence from our previous studies of black flies [10, 13, 21, 22]. *Leucocytozoon* diversity was estimated using the Shannon H′ index, with each *Leucocytozoon* lineage (= cyt *b* haplotype) [14] equal to a species. The Shannon H′ index was calculated in PAST ver. 4.12b [33]. Because each collection was associated with a different number of collectors and durations of collection time, we standardized black fly abundance as the number of adult flies per collector per hour (number of adult flies/collector/hour). The prevalence of *Leucocytozoon* for pooled specimens was estimated in the Epitools Epidemiological Calculators (https://epitools.ausvet.com.au/ppfreqone; accessed 24 April 2024). Minimum and maximum air temperatures were obtained from the Thai National Hydroinformatics Data Center (https://www.thaiwater.net/weather; accessed 13 July 2024) for the closest weather station to the sampling sites. Because generation time of black flies in tropical regions, such as Thailand, is approximately 1 month [34], the minimum and maximum air temperatures were determined for a 1-month period dating from the collection date. Variables (Shannon H′ index, prevalence and minimum and maximum air temperature) were tested for normality and transformed using log10 or square root, if necessary, before regression analysis was performed.

## Results

### Host blood meal and black fly identification

Four blood-engorged females were identified morphologically as *S*. *khelangense*. The COI barcoding sequences of these blood-fed females corroborated the morphological identification with > 99% sequence similarity. Three of the four blood-engorged females were successfully sequenced for host source of DNA in the blood, and all were identical with chicken (*Gallus gallus*).

### Prevalence of *Leucocytozoon* parasites in black flies

In total, 169 samples were positive for *Leucocytozoon* DNA from the 300 individual females of the *S*. *asakoae* complex and 170 pools of *S*. *khelangense*. However, only 152 samples (34 from the *S*. *asakoae* complex and 118 from *S*. *khelangense*) were successfully sequenced (Table 1). The overall prevalence of *Leucocytozoon* parasites in the *S*. *asakoae* complex and in *S. khelangense* was 11.3% and 21.1%, respectively (Table 1). In each collection, the prevalence of *Leucocytozoon* in the *S*. *asakoae* complex varied from 9% at the CM559 and CM556 sites in Chiang Mai province in the northern region to 13% at the CM613 site also in Chiangmai province. The prevalence rates for a location at Ban Pang Bong, Chiangmai province, where specimens were collected in 2019, 2020 and 2023 varied from 9% in 2019 to 13% in 2023 (Table 1).

For *S*. *khelangense*, prevalence rates varied from 13% in the collections from Beung Khong Long, Buengkan province (BK) in the northeastern region to 37% in BK644 in the same province. *Leucocytozoon* prevalence was also high (32%) at a sampling site in Loei province (LO648) in the northeastern region (Table 1). Regression analysis revealed that *Leucocytozoon* prevalence in black flies was not significantly related to black fly abundance or temperature. However, correlation analysis indicated that *Leucocytozoon* prevalence in black flies was significantly and positively related to black fly abundance (*r* = 0.551, *P* = 0.012).

### Diversity of *Leucocytozoon* in black flies

A total of 54 lineages were identified from the 152 cyt *b* sequences obtained in the present study. Among these, 10 (GALLUS06, GALLUS07, GALLUS17, GALLUS18, GALLUS34, GALLUS35, GALLUS37, GALLUS41, GALLUS44, and GALLUS46) were lineages that were present in the MalAvi database, and the remaining 44 were novel lineages found in our study. Nine lineages were found in the *S*. *asakoae* complex in a total of 34 cyt *b* sequences, and 45 lineages were found in *S*. *khelangense* in 118 cyt *b* sequences. The most common lineage was GALLUS17, which was shared by 22 individuals, and GALLUS44, shared by 15 individuals. These common lineages were each specific to a black fly taxon; GALLUS17 was found only in *S*. *khelangense*, whereas GALLUS44 was found only in the *S*. *asakoae* complex. In addition, nine lineages were detected exclusively in the *S*. *asakoae* complex and 45 were found only in *S*. *khelangense* (Additional File 1: Table S1). Analysis of similarity (ANOSIM) of *Leucocytozoon* lineages in the *S*. *asakoae* complex and those of *S*. *khelangense* indicated that these were significantly different (*R* = 0.3556, *P* = 0.0216).

Diversity of *Leucocytozoon*, as measured with the Shannon H′ index, indicated that *S*. *khelangense* (H′ = 3.408) possessed much greater *Leucocytozoon* diversity than did the *S*. *asakoae* complex (H′ = 1.920). In each sampling location, the NK637 population of *S. khelangense* from Nongkhai province in the northeastern region showed the greatest diversity, with 21 *Leucocytozoon* lineages and H′ = 3.044 (Table 2). Multiple regression analysis revealed that *Leucocytozoon* lineage diversity based on the Shannon H′ index was positively related to black fly abundance and negatively related to maximum air temperature (H′ = 4.260 + 0.766Abundance – 0.115Maximum temperature; *F* = 10.135; *P* < 0.001; *df* = 2, 19;* R*^2^_adj_ = 46.5%).

**Table 2:** Black fly abundance, number of *Leucocytozoon* lineages, Shannon H′ index and maximum and minimum air temperature for each collection of the *Simulium asakoae* complex and *S. khelangense* in Thailand

Species/code	Black fly abundance (*n*/collector/hour)	*Leucocytozoon* detected (*n*)	*Leucocytozoon* lineages (*n*)	Shannon H′ index	Maximum air temperature (°C)	Minimum air temperature (°C)
*Simulium asakoae complex*						
CM559	21.0	3	3	1.432	33.3	24.1
CM556	50.7	6	3	1.265	33.1	24.0
CM596	148.2	12	3	0.907	34..3	15.5
CM613	53.0	13	7	1.923	36.8	24.3
*Simulium khelangense*						
NK637	804.0	31	20	3.044	36.2	17.5
UB638	58.5	10	7	2.187	31.7	20.6
BK	268.7	20	13	2.758	32.6	16.6
BK643	82.5	10	7	2.134	36.7	10.1
BK644	719.0	18	7	1.965	36.7	10.1
LO648	337.2	17	11	2.576	33.0	16.3
LO651	241.2	12	9	2.428	32.6	17.8

### Phylogenetic analysis of *Leucocytozoon* in black flies from Thailand

**Fig. 2:**
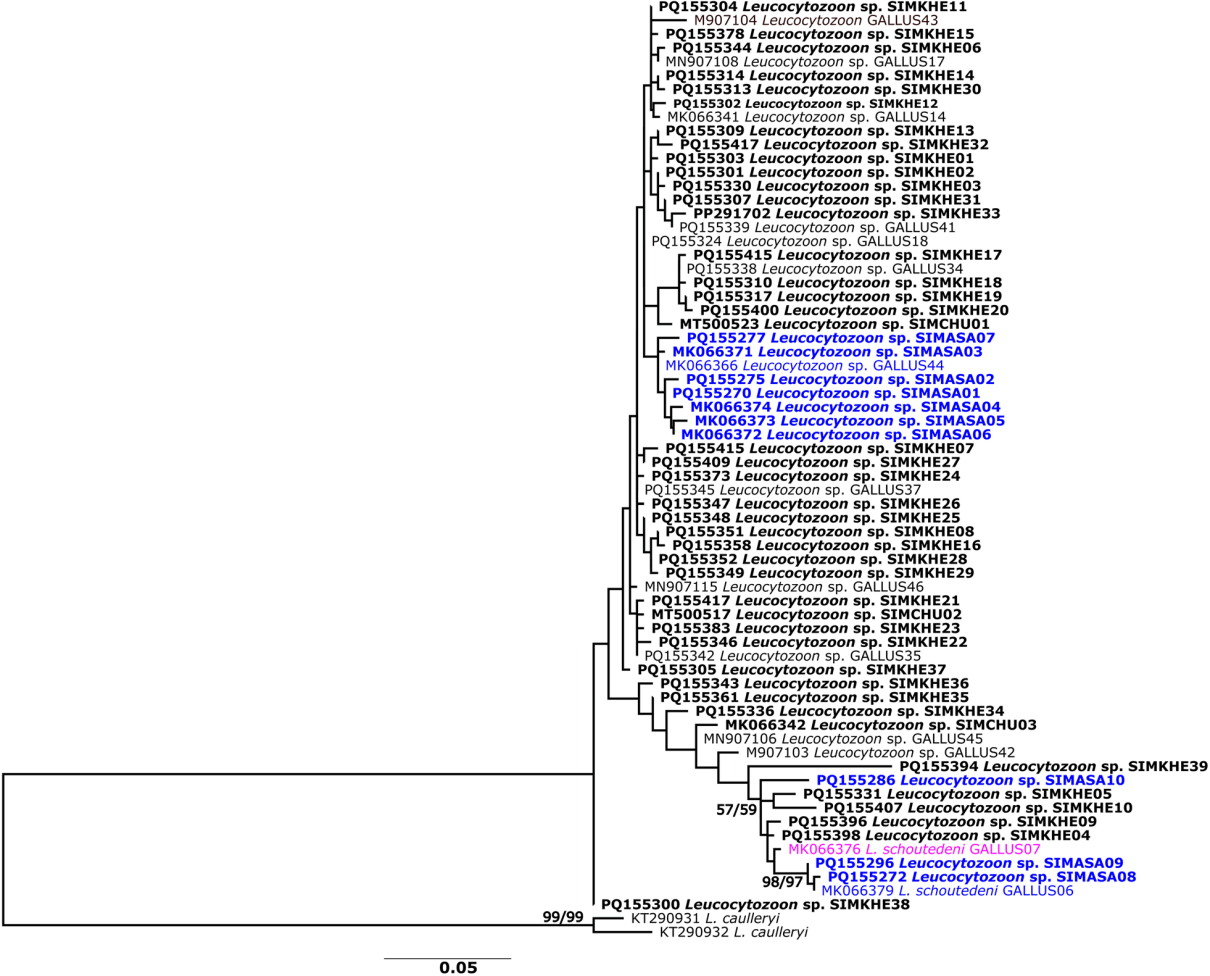
Maximum likelihood tree based on the mitochondrial cytochrome* b* gene sequences of 66 *Leucocytozoon* lineages detected in the *Simulium asakoae* complex (blue text) and *S. khelangense *(black text) in Thailand. The lineage found in both black fly species is indicated in purple. Bold indicates new lineages reported in the present study. Bootstrap values based on 1000 replications for ML and NJ analyses are shown near the branch

The NJ and ML analyses of *Leucocytozoon* lineages in black flies from Thailand revealed similar tree topologies; therefore, only the ML tree is presented here (Fig. 2). The ML tree indicated no major divergent clade. All lineages identified in the MalAvi database as *Leucocytozoon schoutedeni* (GALLUS06, GALLUS07) belonged to a clade that also included two lineages (SIMKHE08 and SIMKHE09) of unidentified *Leucocytozoon*. This is the only minor clade that received high (> 97%) bootstrap support. For *Leucocytozoon* sp., although there was no clear divergent clade, the lineages detected in the *S. asakoae* complex and *S. khelangense* were largely isolated. Only one lineage (SIMASA10) from the *S. asakoae* complex was genetically closer to those from *S. khelangense*.

## Discussion

We report sequences of 44 additional genetic lineages of *Leucocytozoon* in two taxa of black flies (*S. asakoae* complex and *S. khelangense*) in Thailand. All of these belong to the *Leucocytozoon* lineage reported from chickens, except for one that is closest to *Leucocytozoon* reported from owls. This latter lineage was originally reported from a brown hawk-owl (*Ninox scutulata*) in Japan [35] and has also been reported in this bird species in Thailand [19]. Because *Leucocytozoon* lineages are specific to vertebrate hosts [2, 8, 11, 36], identifying the genetic lineages of these parasites can provide a link to the host associations of the blood-sucking vectors [11]. Thus far, the most common blood hosts of the two abundant ornithophilic black fly taxa in Thailand, the *S*. *asakoae* complex and *S. khelangense*, are chickens, although both species also feed on humans [21, 37]. Another avian species that has been recorded as a blood host of *S. khelangense* (= *S. chumpornense* reported by Gomontean et al. [37]) is turkey (*Meleagris gallopavo domesticus*). Thus, finding this black fly carrying the *Leucocytozoon* lineage of owls indicates that *S. khelangense* also feeds on owls; this is the first record of this association for Southeast Asian black flies. The only record to date of wild birds fed upon by Asian black flies is the Japanese rock ptarmigan (*Lagopus muta japonica*). In Japan, at least three black fly species, *Simulium japonicum*, *Prosimulium hirtipes* and *Stegopterna takeshii* (as *Cnephia mutata*), feed on this ptarmigan [38]. Two of these black flies (*S. japonicum* and *P. hirtipes*) as well as *S. uchidai* are possible vectors of *Leucocytozoon lovati* to this endangered subspecies of bird [7].

The authors of a previous study reported a high prevalence (49%) of *Leucocytozoon* in the *S. asakoae* complex collected in January 2018 at Ban Pang Bong in Chiangmai province, Thailand, compared with other sampling sites [10]. We found that *Leucocytozoon* prevalence rates in the *S. asakoae* complex at this location from 2019 to 2023 (9% in 2019, 12% in 2020 and 13% in 2023) were much lower than those in 2018 reported by Jumpato et al. [10]. The high prevalence of *Leucocytozoon* in the *S*. *asakoae* complex collected in 2018 was possibly due to the majority of specimens used for *Leucocytozoon* detection being adult flies resting on vegetation [10], perhaps resting after taking a blood meal from the hosts (e.g. chickens). In this scenario, these specimens had a greater chance of being positive for *Leucocytozoon* than did those swept from the air, which were more likely to have been searching for a host at time of collection.

The prevalence of *Leucocytozoon* found in the present study (11.3% for *S. asakoae* complex and 21.1% for *S. khelangense*) was much higher than rates reported from black flies in Japan (1.6%) [7] but lower than those from Sweden (62%) [36], the USA (46.2% in *S. silvestre*) [8] and Germany (29.4%) [11]. Vectors acquire haemosporidian parasites from vertebrate hosts only via blood-feeding. Therefore, the prevalence of *Leucocytozoon* in black flies depends on its prevalence in the vertebrate host and the probability that the vector and host encounter each other. Prevalence rates of *Leucocytozoon* in Thailand are low in wild birds (2% in raptors [19] and 8.1% in 12 wild bird species from Chiangmai province [20]) but much higher in domestic chickens (18.0–89.5%) [18, 22, 39, 40]. The relatively high prevalence of *Leucocytozoon* in the *S. asakoae* complex and *S. khelangense* compared with its prevalence in wild birds corresponds with the host blood source, primarily domestic chickens [21, 37]. We found a positive relationship between prevalence rates and black fly abundance (*r* = 0.551, *P* = 0.012). This finding agrees with previous reports that the prevalence of *Leucocytozoon* in black flies is associated with environmental factors that promote the production of large populations of these insects [9, 16, 17]. Given the high prevalence (up to 89%) of *Leucocytozoon* in domestic chickens in many areas of Thailand [18], a high abundance of black flies should increase the chance of uptake of the parasite and an increase in its prevalence in the vectors.

At least 48 black fly species are known as vectors of *Leucocytozoon*, and most of these can transmit diverse lineages of *Leucocytozoon* [3, 6–13]. However, some black fly species are specific to a particular parasite lineage. For example, *Simulium annulus* is specific to the *Leucocytozoon* IGRYS1 lineage, perhaps because of a possible host preference, as all analyzed *S. annulus* in northern Sweden fed only on the Eurasian crane (*Grus grus*) despite being collected from geographically distant (200 km) locations [36]. We found variations in the degree of specific vector–parasite associations. Among 66 *Leucocytozoon* lineages in black flies in Thailand, only one was shared between the *S. asakoae* complex and *S. khelangense*. Both taxa also possessed the dominant *Leucocytozoon* lineages that were found in geographically widespread populations but were exclusive to either the *S. asakoae* complex or to *S. khelangense*. Lineages GALLUS17, GALUUS35 and GALLUS37 were the dominant lineages in *S. khelangense* and occurred across a geographically widespread area covering all sampling sites of *S. khelangense*, encompassing > 500 km transects. Similarly, the GALLUS06, GALLUS44 and SIMASA01 lineages were predominant in the *S. asakoae* complex. The latter lineage is also found in a location (Song Khon Waterfall, Phu Ruea, Loei province) [10]) in close geographical proximity (< 12 km) to the lineages of *S. khelangense* (Ban Nong Bua, Phu Ruea, Loei province) [21]). Furthermore, *L. schoutedeni* was also significantly more frequently in the *S. asakoae* complex than in *S. khelangense* (*χ*^2^ = 31.72, *df* = 1, *P* < 0.0001). Because both species feed predominately on chickens, different lineage assemblages in these black flies are unlikely to be a result of different host preferences [36, 41]; rather, different *Leucocytozoon* lineages in the *S. asakoae* complex and *S. khelangense* are possibly related to different ecologies and phenologies of these black flies. The *S. asakoae* complex attains peak abundance in the rainy season [23, 42], whereas *S. khelangense* (= *S. chumpornense* in Pramual et al. [22]) predominates during the hot season [22]. In addition, the *S. asakoae* complex is dominant at higher elevations (> 600 m a.s.l.) compared with *S. khelangense* (mostly < 600 m a.s.l.) [37]. Season and elevation are strongly related to temperature, one of the main environmental factors related to haemosporidian parasite prevalence [16, 17]. Accordingly, we found a significant relationship between diversity of *Leucocytozoon* lineages and temperature. Thus, differences in *Leucocytozoon* lineages in the *S. asakoae* complex and *S. khelangense* might be a result of co-adaptation between vector and parasite in response to the temperature in different seasons and at different elevations. To gain further insight into vector–parasite co-evolution, studies are needed to examine the relationship between black fly haplotypes and those of *Leucocytozoon* lineages [43].

## Conclusions

We found a high diversity of *Leucocytozoon* in black flies of two ornithophilic taxa, the *S*. *asakoae* complex and *S. khelangense*, in Thailand. Our results indicate an association between *Leucocytozoon* lineages and these black flies even though they feed on the same hosts, possibly indicating vector–parasite co-adaptation in response to the environmental conditions of their respective habitats. Further investigation based on the individual haplotypes of the insect vector and the *Leucocytozoon* lineage will be helpful in testing the hypothesis of the vector–parasite co-evolution system [43].

## Supplementary Information

**Additional file 1: Table S1.** Number of each *Leucocytozoon* lineage detected in black flies from each collection site.

## Data Availability

No datasets were generated or analysed during the current study.
